# Repetitive transcranial magnetic stimulation for enhancing motor function after spinal cord injury: a narrative review

**DOI:** 10.3389/fneur.2025.1587060

**Published:** 2025-07-02

**Authors:** Francisco Benavides, Mary Grace Shine, Filip Stefanovic, Robert Chen, Hang Jin Jo

**Affiliations:** ^1^Department of Rehabilitation Science, School of Public Health and Health Professions, University at Buffalo, Buffalo, NY, United States; ^2^Department of Biomedical Engineering, School of Engineering and Applied Sciences, University at Buffalo, Buffalo, NY, United States; ^3^Division of Neurology, Department of Medicine, Krembil Research Institute, University of Toronto, Toronto, ON, Canada

**Keywords:** repetitive TMS, neuroplasticity, spinal cord injury, motor rehabilitation, iTBS

## Abstract

Spinal cord injury (SCI) often leads to disabilities that significantly impact quality of life, highlighting the need for effective rehabilitation strategies. Repetitive transcranial magnetic stimulation (rTMS) has emerged as a promising neuromodulatory approach to enhance neuronal plasticity and promote motor recovery following SCI. This narrative review examines the current state of evidence regarding the therapeutic use of rTMS for motor function recovery after SCI and outlines key methodological considerations to guide future research. To address these aims, we summarize various rTMS techniques and evaluate their overall efficacy in improving motor function in individuals with SCI. Among the fourteen studies reviewed, early rTMS protocols primarily utilized low-frequency stimulation, whereas more recent approaches have adopted higher frequencies and more complex patterned protocols. Despite considerable heterogeneity in stimulation parameters, most studies reported beneficial effects of rTMS, including reduction in spasticity and improvements in voluntary motor function of both upper and lower limbs. These findings demonstrate that rTMS holds promise as an effective tool for SCI rehabilitation, with limited to moderate evidence supporting reductions in spasticity, increased muscle strength, and enhanced functional outcomes. However, many of these findings are derived from small sample sizes, varied protocols, and studies lacking rigorous control conditions. The review emphasizes the need for standardized functional and electrophysiological assessments to systematically evaluate motor outcomes following rTMS interventions. Additionally, larger, well-controlled clinical trials incorporating consistent physical therapy protocols are essential to confirm the efficacy of rTMS.

## Introduction

Non-invasive stimulation of the motor cortex using transcranial magnetic stimulation (TMS) was first reported by Anthony Barker and his colleagues at the Royal Hallamshire Hospital in Sheffield, England (1). The magnetic field produced around the TMS coil can penetrate the scalp and skull and induce an electrical field in the brain area noninvasively (2). This causes current to flow in the brain and excite axons that are near the TMS coil. When the TMS coil is positioned at the cortical representation of a particular muscle, at a strong enough (i.e., above-threshold) intensity, a single TMS pulse can generate a twitch in the contralateral muscle. This muscle twitch, termed motor evoked potential (MEP), can be recorded and monitored online by using electromyography. TMS has been extensively used for the past 40 years to study the motor system with an advantage of eliciting immediate, measurable responses in the muscles. The existence of MEP is a confirmation of an intact anatomical pathway between cortex and muscle, whereas the properties of MEP such as latency and amplitude can provide information about the functional status of the pathway. Such measurements can be particularly informative in an impaired motor system. For example, studies using TMS of the primary motor cortex (M1) showed that the MEPs in individuals with spinal cord injury (SCI) have different characteristics compared to uninjured individuals, including decreased amplitude, longer latency and higher threshold (3, 4). Further, MEP parameters have been shown to predict the neurological and functional outcomes after SCI (5–7).

TMS can be applied using consecutive stimuli at specific interstimulus intervals or in a patterned form. Such protocols are referred to as repetitive TMS (rTMS) and may have modulatory effects on cortical excitability that outlast the stimulation period. When rTMS is applied on the M1, changes in cortical excitability can be easily measured by assessing the amplitude of MEPs. Generally, rTMS protocols with frequencies below 1 Hz tend to produce an inhibitory effect (8), while frequencies of 5 Hz or higher primarily result in excitatory effects in the amplitude of MEP (9). These excitatory or inhibitory effects of rTMS are thought to be related to increases or decreases of synaptic efficiency, such as long-term potentiation (LTP) or long-term depression (LTD) (10). rTMS protocols require specific stimulators capable of maintaining the same stimulus output at such brief interstimulus intervals and often are limited in the maximum stimulation intensity output due to the capacity of the machine. Over the past 30 years, rTMS protocols have evolved significantly with advancement in technology. New devices now allow the use of higher frequencies and intensities, as well as programable pulse sequences, along with customizable pulse shape, polarities and durations.

The use of rTMS has been increasing dramatically since it was first approved by Food and Drug Administration (FDA) in 2008 for major depressive disorder. Since then, the FDA approved rTMS treatment for several other conditions such as migraines, obsessive-compulsive disorder, and smoking cessation. Additionally, the efficacy of rTMS protocols has been extensively studied in numerous conditions with motor impairments including stroke, Parkinson’s disease, essential tremor, and dystonia to improve motor function (11–13), however, its applications in individuals with SCI have been relatively limited. Given that extensive brain reorganization occurs in the neural circuits of motor-related cortical areas following SCI (14, 15), LTP- or LTD-like effects may play a crucial role in restoring spared motor networks, ultimately enhancing motor function after SCI. Indeed, several rTMS protocols applied to individuals with SCI have shown promising results. In this review, we will briefly summarize the different rTMS techniques and their effects on motor functional improvement after SCI. Based on the currently available evidence, this review addresses the following key questions: What is the current state of evidence regarding the therapeutic use of rTMS for motor function recovery after SCI, and what methodological considerations should guide future studies?

## Methods

In this review, we summarize 14 articles that examined the effects of rTMS on the motor functional changes in individuals with SCI (Table 1). We excluded case reports and studies that combined rTMS with other types of noninvasive stimulation, such as transcutaneous spinal stimulation or transcranial direct current stimulation. For each study, we summarized key rTMS parameters, including stimulation frequency, train duration, intertrain interval, total pulse count, coil type (e.g., figure-of-8, double cone, circular), and stimulation intensity relative to motor thresholds (resting or active). Additionally, we documented outcome measures such as spasticity reduction, muscle strength, motor functional improvements (e.g., gait, hand function), and neurophysiological changes (e.g., MEP, reciprocal inhibition). Lastly, based on the reviewed literature, we discussed the mechanisms of action, optimization of rTMS protocols, safety considerations, and ongoing rTMS studies in individuals with SCI.

**Table 1:** Summary of reviewed articles.

Author	Year	Coil type	Pulse type	Stim. frequency (Hz)	Targeted cortex	Intensity	Sham	Rehabilitation	N*	ASIA	Injury level	Outcomes
Belci et al.	2004	Circular	Paired Monophasic	10	Hand M1	90% RMT	Yes	No rehabilitation.	4	D	C5	9HPB test showed improved hand function.
Kumru et al.	2010	Double Cone	Biphasic	20	Leg M1	90% RMT of biceps brachii	Yes	No rehabilitation.	14	C, D	C4-T12	Improved MAS score and reduction of frequency in spasm events.
Kuppuswamy et al.	2011	Figure of 8	Biphasic	5	Leg M1	80% AMT (at 10% MVC)	Yes	No rehabilitation.	15	A-D	C4-C8	No significant changes in ASIA scores. Improved score on specific functional arm movement test.
Benito et al.	2012	Double Cone	Biphasic	20	Leg M1	90% RMT of biceps brachii	Yes	Standard SCI Rehabilitation, five hours of therapy, ground gait training after TMS sessions.	10	D	C4-T12	Improved MAS and LEMS scores.
Nardone et al.	2014	Double Cone	Biphasic	20	Leg M1	90% RMT of biceps brachii	Yes	No rehabilitation.	9	C, D	C5-T10	Lower limb spasticity reduced.
Gomes-Osman et al.	2015	Figure of 8	Biphasic	10	Hand M1	80% RMT of biceps brachii	Yes	Repetitive task practice (RTP).	11	C, D	C6 median	No significant changes in Jebsen-Taylor Hand Function Test and Pinch and Grasp force.
Alexeeva et al.	2015	Figure of 8 and Cone	Quadro (4 pulse trains) monophasic	250–500	Hand and Leg M1	80–90% RMT	No	Targeted individualized exercises.	3	B, C, D	C5, C6, C7	Improvements on the Purdue Pegboard Dexterity test (*n* = 2) and the Treadmill Walking Speed test (*n* = 1).
Kumru et al.	2016	Double Cone	Biphasic	20	Leg M1	90% RMT lowest threshold of APB, FDI, BB	Yes	Lokomat training 30-45 min.	15	C, D	C3-T12	No change in MAS score. Improvements were seen in leg muscle motor strength and gait.
Nardone et al.	2017	Figure of 8	Biphasic	5 and 50	Leg M1	80% AMT or 50% MSO if no AMT	Yes	No rehabilitation.	10	C, D	C5-T8	MAS score reduced for 1 week.
Gharooni et al.	2018	Circular (90 mm)	Biphasic	5 and 50	Leg M1	80% RMT by visual inspection of upper limb muscles	Yes	No rehabilitation.	10	B-D	C3-C6	MAS score was reduced for upper limbs. No motor improvement of upper or lower limbs observed.
Mendonca et al.	2021	Figure of 8	Biphasic	1 and 10	Leg M1	90% RMT of a hand muscle	Yes	No rehabilitation.	11	C, D	T1-L1	No change in MAS score.
Wincek et al.	2021	Circular (120 mm)	Biphasic	15–25	Hand M1 bilateral	70–80% RMT for either hand or leg muscles	No	Kinesiotherapy 4–5 h, five days a week.	26	C, D	C2-C7 T1-T12	Decreased EMG involuntary activity in hand muscles. Increase in voluntary contraction in both hand and leg muscles.
Krogh et al.	2022	Double Cone	Biphasic	20	Leg M1	100% RMT of a hand muscle	Yes	Lower limb resistance training or physical therapy, twice a week for 4 weeks.	10	A-D	C2-L2	LEMS showed significant improvement. No changes in gait function.
Kesikburun et al.	2023	Figure of 8	Biphasic	20	Leg M1	110% RMT of a hand muscle	Yes	30 min of lower extremity strengthening and 30 min of overground walking and balance exercises	13	C, D	C-T	Significant improvement in walking speed and LEMS. No changes in MAS.

Active Motor Threshold (AMT), Cortical Silent Period (CSP), Lower Extremities Motor Scores (LEMS), Modified Ashworth Scale (MAS), Nine-hole peg board (9HPB), Spinal Cord Assessment Tool for Spastic Reflexes (SCATS), Spinal Cord Injury (SCI). *N = The number of participants with SCI who underwent active rTMS protocols.

### Application of rTMS

**Figure 1:**
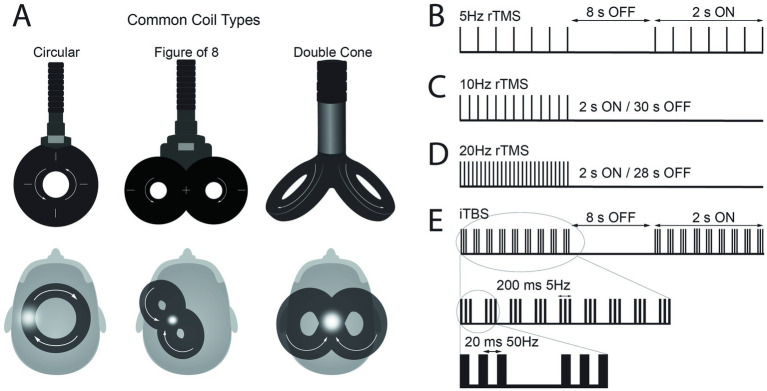
**(A)** Schematic representation of common types of transcranial magnetic stimulation (TMS) coils and their position over the scalp to apply repetitive TMS (rTMS) protocols over M1. The white arrows illustrate the direction of current flow in the coil, which induces an electrical current in the brain flowing in the opposite direction. The white dots represent the hand area of motor cortex for circular and figure-of-8 coils and leg area for double cone coil. **(B–E)** Representation of pulse frequencies and intervals commonly used in rTMS protocols with set frequency and the intermittent theta burst stimulation (iTBS) protocol.

Several aspects of the electromagnetic fields generated and applied near the brain have an impact on modulatory effects on neuronal connectivity. One key aspect is the focality of the magnetic field, which is directly linked to the shape of the electromagnetic coil that generates the field (16). Thus, several shapes of TMS coil have been designed for different purposes (Figure 1A). Circular shapes were commonly used in the early stages of development of TMS. Ultimately, an array of two circular coils, configured in a figure-of-8 shape, was designed with electrical current flowing simultaneously in each wing but in opposite directions to enhance the focality at its center compared to the single circular coils (17, 18). Double cone coils have a similar design to figure-of-8 coils with the two circular coils, but they are placed in an angle (30 to 45 degrees). This design has the advantage of generating a stronger, larger field, that when applied to the brain can reach relatively deeper structures. Thus, double cone coils are often used to stimulate the motor representation of leg muscles located deeper at the midline of the brain in the interhemispheric fissure. Additionally, double cone coils can provide more reliable cerebellar stimulation applied to the back of the head compared to the figure-of-8 coils because the cerebellum is located deep (19). However, there is a tradeoff between electric field depth of penetration and focality (20); overall, double cone coils are better suited for stimulating deeper structures while figure-of-8 coils provide more focal stimulation.

rTMS can modulate cortical excitability, either increase or decrease, and the effect depends on the frequency of stimulation (21, 22). The frequency of the pulses can be broadly classified as high (>1 Hz) or low (1 Hz or lower) frequency. High frequency rTMS typically produces a reduction in cortical inhibition and an increase in MEP size whereas low frequency rTMS often produce a reduction in cortical excitability and a decrease in MEP size (21). New micro circuits and more efficient electrical components have allowed programing more complex patterned rTMS protocols with more than one frequency. An example is the theta burst stimulation (TBS) protocols. TBS includes pulse bursts of three stimuli at 50 Hz (i.e., 20 ms between each stimulus), which is repeated at intervals of 200 ms (i.e., 5 Hz). There are two different ways that TBS is typically applied; first, during continuous TBS (cTBS), TBS is delivered continuously for a total of 600 pulses (~40 s). cTBS tends to decrease the cortical excitability. Second, TBS can be applied for 2 s, with 8 s inter-burst-intervals for a total of 600 pulses (~3 min). This protocol called intermittent TBS (iTBS) produces an increase in the cortical excitability (23). While the above-mentioned aftereffects of rTMS protocols have been increasingly documented in studies, there has also been a consistent observation of large variability in rTMS outcomes measures (24–27), resulting in often contradictory findings (28, 29).

## Results

### Therapeutic effect of rTMS on motor function after SCI

The use of rTMS to enhance motor function in individuals with SCI has grown over the past decade (30, 31). Studies have examined the effects of rTMS applied to the M1 using different stimulation parameters, such as frequency and intensity, and have reported varied results on motor function in individuals with SCI. One of the earliest reports was by Belci et al. in 2004 where they used the adapted paired monophasic pulses to achieve higher frequencies and reported hand functional improvements on 4 participants with SCI at C5 level classified as AIS (American Spinal Injury Association Impairment Scale) D (32). They tested right hand functional scores and reported positive effects lasting up to 2 weeks after the TMS intervention. They used a monophasic magnetic stimulator with a circular coil placed over the vertex. The stimulus intensity was 90% threshold to elicit MEPs in hand muscles, with the current flowing clockwise to activate the left motor cortex. The participants received 5 days of double pulses of rTMS over the occipital cortex (sham treatment) followed by 5 days of double pulses of rTMS over the motor cortex (real treatment). Double pulses were applied at 100 ms interval (10 Hz) every 10 s (0.1 Hz) for a total of 360 double pulses (720 pulses in total) over 1 h. The authors reported improvement in hand function with results of nine-hole peg board test and reduced intracortical inhibition estimated from cortical silent periods measured with EMG.

In many recent rTMS protocols, rTMS is delivered in 2 s trains, with inter-train intervals of 8 to 30 s during which TMS is not applied. First, Kuppuswamy and colleagues reported the effect of 5 consecutive rTMS sessions using trains of pulses of 2 s duration at 5 Hz frequency with inter-train interval of 8 s (Figure 1B) for a total of 15 min in individuals with SCI (33). TMS was delivered at an intensity of 80% active motor threshold (AMT) with a figure-of-8 coil over M1 representation of one of the muscles in the upper extremity. Fifteen individuals with cervical SCI (AIS levels of A, B, C, and D) were included. The study used a randomized sham-controlled cross-over design. Sham protocol used a circular sham coil over the vertex delivering 5% of the 80% AMT intensity. The results showed improvements on upper limb motor function measured by Action Research Arm Test (ARAT) after rTMS protocol compared to baseline.

Gomes-Osman and Field-Fote reported the effect of 10 Hz rTMS protocol in 11 participants with cervical SCI classified as AIS C or D (34). In this study, 10 Hz-rTMS was delivered in 2 s trains with an intertrain interval of 30 s for a total of 800 pulses (Figure 1C). TMS was applied to M1 using a figure-of-8 coil placed tangential to the scalp approximately 5 cm lateral to the vertex with a posterolateral direction at an angle of 45° to the midsagittal line, with an intensity set at 80% of the biceps resting motor threshold (RMT). The study utilized a crossover design that included three sessions of 10 Hz rTMS and three sessions of sham stimulation. Both rTMS and sham stimulation were followed by repetitive motor training. The sham stimulation protocol used electrical stimulation over the scalp to mimic the sensation of rTMS. The authors reported that rTMS sessions were associated with larger effect sizes compared with sham stimulation sessions for improvement in Jebsen-Taylor hand function test and grasp strength, however, there were no statistical between-condition differences. In another study with 10 Hz rTMS protocol, Mendonca et al. applied TMS at 90% of the first dorsal interosseous (FDI) RMT via a figure-of-8 coil to leg M1 in thoracolumbar SCI classified as AIS C or D (35). A 10 Hz-rTMS was administered in 4 s trains with a 28 s intertrain interval, delivering a total of 1,600 pulses. The study reported no changes in the Modified Ashworth Scale (MAS) scores for the lower limb muscles.

In the next set of studies, rTMS was utilized at a higher frequency, 20 Hz, with 2 s trains and inter-train intervals of 28 s (Figure 1D). The effect of this protocol in individuals with SCI was first reported by Kumru and colleagues (36). They reported improvements of spasticity after 5 sessions of 20 Hz-rTMS in 15 individuals with SCI. Participants in this study had a SCI at C4-T12 levels classified as either AIS C or D, with their time since injury ranging from 2 to 17 months. Individuals with a MAS score greater than 1.5 were included in this study. The intervention protocol was 5 daily sessions of rTMS delivered over the vertex with a double cone coil (wings 110 mm in diameter). The 20 Hz rTMS protocol was applied for a total of 1,600 pulses over 20 min. The intensity of the stimulation was set to 90% RMT of the right biceps brachii muscle. Participants were randomized into sham stimulation or real rTMS groups. The sham stimulation protocol placed the double cone coil on subject’s head without triggering and triggered a figure-of-8 coil under the subject’s pillow. After rTMS session, their results showed a significant reduction of spasm frequency and severity compared to the baseline condition, but this change was not observed after sham sessions.

In a subsequent study, Benito et al. reported the effect of a 20 Hz rTMS protocol on the lower extremity motor score and gait in individuals with SCI (37). TMS was applied over the leg representation of the motor cortex with a double cone coil over the vertex at an intensity of 90% RMT of the upper limb muscle with the lowest motor threshold. The rTMS parameters and sham stimulation protocols were consistent with those used in the previous study (36). However, the total number of pulses per session was slightly increased to 1800 and the number of sessions was extended to 15 daily sessions. Seventeen individuals with cervical or thoracic SCI (AIS D) were included in the study. Time since injury did not exceed 1 year in all participants (3–12 months). The results showed improvements in strength of lower limb muscles as well as walking speed measured by lower extremities motor score and 10-meter walk test. The study also reported a significant improvement in the MAS, i.e., a decrease in spasticity. No improvement was observed following sham stimulation.

To further investigate the neurophysiological mechanisms of the 20 Hz rTMS protocol, Nardone and colleagues utilized the previously reported rTMS protocol (36) and measured changes in spasticity and reciprocal inhibition in individuals with SCI (38). This study included 9 participants with cervical or thoracic SCI classified as AIS C or D. Their results showed that multiple sessions of rTMS over the M1 reduced spasticity in subjects with SCI and restored the impaired excitability in the disynaptic reciprocal inhibitory pathway.

Kumru and colleagues conducted another study using 20 Hz rTMS and combined it with robotic assisted gait training in individuals with SCI who were less than 6 months post-injury (39). This study included 31 participants with cervical or thoracic SCI (AIS C or D) and randomly assigned them to receive 20 sessions of Lokomat gait training with either rTMS or sham rTMS, and sham protocols were implemented as described in their previous studies (36, 37). The combination of rTMS with Lokomat gait training led to greater clinical improvement in lower extremity motor strength compared to sham stimulation. During the follow-up, more subjects (71.4%) could perform 10-m walk test in rTMS group than in sham group (40%), but the differences did not reach the level of significance.

More recently, Krogh and colleagues applied 20 Hz rTMS as reported in a previous study by Benito et al. through a double cone coil (37) but set the stimulation intensity higher at 100% RMT of an intrinsic hand muscle (40). The study included 19 participants with SCI (cervical to lumbar injuries with AIS A, C, and D) and they were randomly assigned to 20 sessions of lower limb motor training combined with either rTMS or sham stimulation. The results showed greater increase in lower extremity motor scores in rTMS group compared to sham group. However, functional measurements such as 10-meter walk test and Timed Up-and-Go test did not show the effect of time or stimulation type. Additionally, this study reported a seizure as an adverse effect during rTMS in a young participant with no personal or family history of epilepsy (40).

Kesikburun et al. applied 20 Hz rTMS to leg M1, as reported by Kumru et al. (36), but used a figure-of-8 coil and increased the stimulation intensity to 110% RMT of an intrinsic hand muscle (41). Twenty-eight participants with SCI (cervical to thoracic injuries with AIS C, and D) were randomly assigned to either sham or real rTMS group. After 15 sessions of stimulation combined with gait training, the real rTMS group showed significant improvements in walking speed and LEMS score, with these changes maintained at 2-week follow-up. However, no changes were observed in MAS scores at any time point.

Wincek and colleagues applied the high frequency rTMS (15–25 Hz), for the first time, bilaterally in individuals with SCI (42). In each session, 800 biphasic pulses were delivered to each hemisphere (1,600 pulses in total), with stimulation for 10 min followed by 10 min of non-stimulation. A maximum of 15 sessions of rTMS was applied within 5 months on average combined with exercise training. TMS was administered using a circular coil at 70–80% RMT for either hand or leg muscles. As in the 20 Hz rTMS protocols reported above (36, 37), this study used 2 s trains (15–25 Hz) with 28 s intertrain intervals. Notably, the frequency of rTMS was individually determined through algorithms designed in this study based on the physiological outcomes; the frequency of rTMS was adjusted higher (from 15 up to 25 Hz), depending on the MEP results and EMG recordings during voluntary contraction. The study reported results of 51 participants with cervical or thoracic SCI classified as AIS C and D (26 received rTMS). The investigators reported significant changes in physiological measurements in the group that received exercise training with rTMS (*n* = 26), compared to the group that received exercise training without stimulation (*n* = 25). After rTMS sessions, there was increased MEP amplitude and decreased involuntary activities at rest in EMG recordings of hand and leg muscles.

Nardone and colleagues reported the effects of another high frequency rTMS protocol, the iTBS, on spasticity in participants with SCI (43). During iTBS, a burst of 3 stimuli at 50 Hz (i.e., 20 ms between each stimulus) was repeated every 200 ms (i.e., 5 Hz) for 2 s, with 8 s-intervals, for a total of 600 pulses (~200 s) at an intensity of 80% AMT (Figure 1E). The stimulation was applied with a figure-of-8 coil over the leg representation of the M1. Ten participants with cervical or thoracic injuries (AIS C or D) were included in this study and underwent 10 sessions of rTMS or sham stimulation. The sham stimulation protocol applied stimulation at 15% of maximal stimulation output with the coil rotated 90° to ensure no current was induced in the brain. The iTBS protocols produce a consistent LTP-like effect, causing a prolonged increase of motor cortex excitability (23) and have been more extensively studied in stroke patients (44). Similar to those results, subjects with SCI showed increased corticospinal excitability, indicated by larger MEP amplitudes, after rTMS sessions. Additionally, spinal excitability measured by the H/M amplitude ratio also increased. Clinical measurements of spasticity, such as MAS and the Spinal Cord Injury Assessment Tool for Spasticity (SCAT), also significantly improved after rTMS sessions.

Gharooni and colleagues also reported the effect of iTBS in individuals with SCI (45). They applied iTBS using a 90 mm circular coil placed over the vertex at 80% RMT. RMT was determined by visually inspecting muscle twitch responses in the upper limbs. Ten individuals with cervical SCI (AIS B, C, or D) underwent 10 sessions of rTMS and 10 sessions of sham stimulation in a randomized order with a 2-week washout period. The sham protocol was to rotate the coil 90° as described in Nardone et al. (43). Although there were some improvement in upper and lower limb motor scores and MAS, no statistical differences were reported between iTBS and sham stimulation groups in this study.

To date, Alexeeva and Calancie have implemented the highest frequency rTMS protocol for individuals with SCI, referred to as QuadroPulse TMS (46). QuadroPulse consists of trains with four pulses at interpulse intervals ranging from 2 to 4 ms (i.e., 250 to 500 Hz) and an intertrain interval of 5 to 6 s. The number of trains delivered within a single daily session was either 250 or 360 (i.e., approximately 23 or 33 min). The QuadroPulse was applied at 80 to 90% RMT via a figure-of-8 coil to stimulate the hand representation of M1 or a double cone coil to stimulate the leg representation of M1 over vertex. Three participants were included in the proof-of-concept study; all three participants had cervical spinal cord injuries, classified as AIS B, C, and D. The authors reported functional improvements in both hand dexterity and treadmill walking speed after 5 days of QuadroPulse TMS.

## Discussion

Overall, rTMS has been shown to be a promising tool for SCI motor rehabilitation in the above articles. In individuals with SCI, rTMS protocols effectively reduced spasticity (5 out of 8 studies), increased muscle strength in the upper and lower limbs (5 out of 8 studies) and resulted in functional improvements (5 out of 8 studies). Future studies applying rTMS in SCI could benefit from the considerations outlined below.

### Mechanisms of action

The understanding of the mechanisms driving these functional improvements remains limited, as only a few studies have incorporated physiological measurements (32, 38, 42, 43). Future research needs to focus on these underlying mechanisms, as this could potentially enhance the efficacy of rTMS protocols. While physiological measurements will be valuable in human studies, animal models of SCI can also offer crucial insights into the physiological mechanisms involved in plasticity. For example, recent study on iTBS protocols in animal models of SCI have improved our understanding of the mechanism of neuroplasticity following iTBS. Marufa and colleagues tested the effect of iTBS protocol using a rodent model of thoracic SCI (47). The study applied a clip compression around the spinal cord at T10 to produce hindlimb impairments in an SCI rat model with mild, moderate, and severe severities. The iTBS protocol was identical to human studies; trains of three pulses at 50 Hz repeated at a frequency of 5 Hz for 2 s with 8 s-interval for a total of 600 pulses. The iTBS intensity was set at 80% of the RMT, which was determined during muscle relaxation induced by anesthesia. The iTBS protocol was administered 10 times in total for 2 weeks. Their sham protocol was identical to the actual stimulation, except that the coil was placed 8 cm above the rat’s head. The results of this study showed an increase in MEP amplitude in the hindlimb muscles of the mild and moderate SCI groups compared to baseline and sham sessions. However, this effect was not observed in the severe SCI group. While it is challenging to extrapolate animal models of SCI to humans, they can offer valuable insights into changes in the neurochemical components in the cortical and spinal tissue; the growth-associated protein-43 (GAP-43) was significantly increased following 2 weeks of iTBS compared to the sham group. The expression of this protein is related to axonal regeneration in mature axons, when upregulated GAP-43 promotes reinnervation and nerve sprouting in lesioned tissues.

### Optimization of rTMS protocols

Although previous rTMS studies in individuals with SCI have demonstrated promising results across a range of protocols, there remains no standardized approach regarding threshold assessment, stimulation intensity or optimal pulse number. Direct comparisons of different rTMS parameters are needed to identify the most effective protocol in this population. Future studies need to preliminarily evaluate the most promising protocols before going on to multicenter studies with large cohorts of patients. Additionally, it would be valuable to investigate the recent advancements in existing protocols in SCI. A recent study provided a potential to optimize plasticity induction of iTBS protocol by constraining the brain state with a behavioral task during stimulation (48). They demonstrated that parietal iTBS during a behavioral manipulation, i.e., performing a grasping task concurrently, increased the corticospinal excitability and improved motor performance relative to iTBS during rest. Further, combination of TMS with electroencephalography (EEG) has provided an emergent method to apply rTMS more effectively (49). Recent protocols such as closed-loop EEG-rTMS can identify specific EEG components and trigger rTMS to time the delivery of rTMS relative to an endogenous brain state (50). These newer approaches have the potential to increase the efficacy of rTMS protocols compared to conventional methods in individuals with SCI.

While the variability of effects of rTMS protocols on corticospinal excitability has been acknowledged in previous studies in control subjects, it has not been addressed in any of the reviewed articles in SCI. Understanding this variability in rTMS responses among individuals with SCI is essential for its therapeutic applications. Further, developing biomarkers to predict the efficacy of rTMS in individuals with SCI will allow identification of individuals who will show significant functional changes with rTMS protocols and facilitate its clinical application. Future studies could investigate whether changes in corticospinal excitability during the first session predict an individual’s response to multiple rTMS sessions. Factors such as the severity of SCI, time since injury, and functional level may influence how a person responds to rTMS protocols. Incorporating additional techniques, such as brain and spinal cord imaging, EEG, and machine learning, may help predict the long-term efficacy of the protocols.

Neurostimulation approaches such as epidural (51–54) or transcutaneous spinal stimulation (55) have shown to facilitate the effect of exercise trainings in SCI. Similarly, to further improve the outcomes of rTMS protocols, combining them with rehabilitation strategies has become a prominent area of interest. Among the 14 articles reviewed, 7 studies incorporated at least one type of rehabilitation strategy. However, we did not observe any clear trend indicating that adding motor training to rTMS protocols is beneficial for improving motor function, muscle strength or spasticity. This result may be attributed to the wide variability in the rehabilitation protocols used. Future studies should explore whether the potentiating effect of motor training depends on rehabilitation strategies tailored to address the specific treatment goals. Additionally, determining the minimum number of sessions required to observe motor improvements with rehabilitation exercise is crucial for designing future experiments that combine rTMS protocols with rehabilitation exercises.

### Safety considerations

Among the 14 reviewed studies, one study reported a seizure associated with rTMS in an individual with SCI (40). Notably, this study used a double cone coil and a relatively higher intensity (100% RMT of a hand intrinsic muscle) compared to other studies. Other studies reported mild and transient side effects, including facial muscles twitching during the first session of active stimulation (36, 37, 39, 40), mild drowsiness (35, 40, 46), neck pain (35), mild headache (39), and tingling sensations in the scalp (40). Four studies explicitly stated that no adverse event or side effects occurred (34, 41, 43, 45), while the remaining four studies did not report any safety-related information (32, 33, 38, 42).

There is a tendency of using high stimulation intensity and frequency in more recent studies. Given that stimulation intensity is determined based on an individual’s motor thresholds, which are typically elevated following SCI (56, 57), it would be beneficial for future rTMS studies involving individuals with SCI to report both normalized intensity relative to motor threshold (e.g., %AMT or %RMT) and the actual stimulation intensity expressed as a percentage of the maximal stimulator output (MSO). This would help establish guidelines for safe stimulation parameters in individuals with SCI.

### Ongoing rTMS studies in SCI

Several recently completed and ongoing clinical trials listed on ClinicalTrials.gov have applied rTMS in individuals with SCI, reflecting growing interest in its potential as a therapeutic modality. These studies have begun to address key methodological gaps highlighted in earlier work. For example, NCT06248476 compares two rTMS protocols, iTBS and high-frequency rTMS, for improving ambulation and lower limb motor function in individuals with chronic SCI. Other trials combine rTMS with specific forms of motor training, such as high-intensity resistance training (NCT03690726), body-weight supported treadmill training (NCT03394560), and robotic gait training (NCT06188131) to enhance lower limb recovery. Additionally, NCT05333770 evaluates the feasibility and efficacy of high-frequency rTMS in subacute SCI. Ongoing trials include NCT06464744, which is testing 15 sessions of rTMS with a 12-month follow-up to assess upper and lower limb motor outcomes, and NCT06247904, which investigates 2 weeks of bilateral high-frequency rTMS. Notably, several of these studies employ rigorous sham-controlled, double-blind designs and incorporate long-term follow-up periods to assess the effects. Collectively, these trials will provide valuable data on safety, optimal stimulation parameters, and clinical efficacy of rTMS in SCI rehabilitation.

## Conclusion

rTMS has demonstrated promise as an effective tool for SCI rehabilitation, with limited to moderate evidence showing reductions in spasticity, increases in muscle strength in both upper and lower limbs, and enhanced functional outcomes. While these findings highlight the potential beneficial effects of rTMS, many of these findings are derived from small sample sizes, heterogeneous protocols, and studies lacking rigorous control conditions. Future studies should address the need for standardized functional and electrophysiological assessments to systematically evaluate motor function outcomes. Larger clinical trials, including control groups with consistent physical therapy protocols, are essential to confirm these effects and support broader clinical implementation.
